# A Complete Course of Meteorology

**Published:** 1845-04

**Authors:** 


A complete Course of Meteorology. By *L. F. Kaemtz*, Professor of Physics at the University of Halle; with Notes, by *Ch. Martins*, Supernumerary Professor of Natural History to the Faculty of Medicine, Paris, and an Appendix by *L. Lalanne*, Translated with Notes and Additions, by *C. V. Walker*, &c. &c. Fifteen Plates, pp. 598. London, 1845. Bailliere.

Meteorology is one of those branches of natural science that especially demands the study of the medical man, from the well-known and acknowledged influence which the varying conditions of the atmosphere have on the production and diffusion of many diseases. During the present century the relations, that unquestionably exist between the origin of certain epidemics and an oscillatory or *feverish*, so to speak, state of barometric phenomena, have been far too much overlooked; and much is it to be desired, that more attention had been uniformly paid to the subject. That the character and type of many diseases—those more especially of the class Pyrexiæ—are so materially modified by the changing conditions of the air around us, as to require a very different treatment in different years, will be admitted by all practitioners of experience. The writings of Sydenham, more particularly, have taught physicians to appreciate the importance of what he calls the "medical constitution" of different seasons. The subject is one of great practical interest, although it must be confessed that allusion is rarely made to the operation of this agency in modern publications.

The present work contains a large amount of information on most of the topics that are usually considered to appertain to Meteorological Science. The first chapter is occupied with *Considerations on the Range of Temperature in general;* the second treats *Of Winds;* the third of *Aqueous Meteors;* the fourth of the *Distribution of Temperature on the Surface of the Globe;* the fifth of the *Weight of the Atmosphere;* the sixth of the *Electric Phenomena of the Atmosphere;* the seventh of the *Optical Phenomena of the Atmosphere;* the eighth of *Auroræ Boreales;* and the ninth and last, of what are called *Problematical Phenomena*—such as showers of blood, of sulphur, animals, &c.; shooting stars, and meteoric stones, &c. &c.

In selecting our extracts, we shall endeavour to keep in view those meteorological subjects that have a more or less immediate reference to animal life, more especially to that of man.

After giving a series of interesting tables to shew the *mean temperature of the atmosphere* at all hours of the day, during the twelve months of the year, as observed at four different places, viz. Halle, Gottingen, Padua, and Leith, our author makes the following instructive remarks:—

"These tables shew that there is a *maximum* and a *minimum* of temperature on each day. The *minimum* occurs some little time before the rising of the sun, the *maximum* about two o'clock in the afternoon; a little sooner in winter and a little later in summer. The majority of philosophers admit that the moment of the rising of the sun is that at which the temperature is the lowest; but if we deduce from observation a formula, independent of the trifling errors of reading, which are almost inevitable, we shall find that the *minimum* occurs about half an hour before the rising of the sun, at the time when this body is yet 12° beneath the horizon. This rule, which is only applicable to our climates, varies in different seasons. In autumn and in winter the *minimum* coincides with a depression of 18° below the horizon, and in summer with 6° only.

"When the sun is above the horizon, it acts upon the earth and the lower strata of the atmosphere with greater power, as its angular height is greater. One portion of this heat penetrates into the soil, the other is lost by radiation towards the atmosphere and celestial space. Before mid-day the earth receives, in every instant of time, a quantity of heat, exceeding that which it loses by radiation; and its temperature is raised. This effect continues also for some time after the sun has passed the meridian, hence it follows that the maximum takes place some hours after the time of noon. While the sun is sinking toward the horizon, its action becomes less powerful, and the loss by radiation exceeds the gain by absorption. The heat diminishes the more rapidly as the sun is nearer setting. As soon as it has disappeared, the calorific source no longer existing, all the acquired heat radiates towards celestial space, the temperature falls, and would fall still lower, if the portion of the heat, which had penetrated into the superficial layers of the soil, did not return to the surface, by virtue of the conducting power of the earth. The lowering of temperature continues until morning announces the return of the sun, which again heats the regions that it illuminates." 20.

As the warmest time of the day is about an hour or two after noon, (when the sun has attained his highest elevation,) so the hottest month of the year is July, or that which succeeds to the Summer Solstice. The reason of this well-known fact is that, in the course of the preceding months, the air and earth have already become warmed, so that the mere continuance of the same thermometric temperature will have the effect of raising it by a few degrees. Moreover the nights, at this period of the year, are very short; and the earth therefore loses comparatively but little caloric by radiation during the absence of the sun.

The mensual heat falls at first slowly in August, then rapidly in September and October, and descends to its *minimum* in the middle of January. From that period, its rise is slow until the beginning of April, in which month, and in May, it becomes much more rapid; it then goes on less rapidly to the end of July, when it attains its *maximum*. The amount of difference between the *maximum* and *minimum* degrees of temperature is always less in small islands, and on the western shores of Continents, than elsewhere.

*Winds.*—We need scarcely say that these phenomena are always caused by a disturbance in the equilibrium of the atmosphere, in consequence of differences of temperature in different regions, or localities.

" *If two neighbouring regions are unequally heated, there is produced in the upper strata, a wind blowing from the hotter to the colder region; and, at the surface of the soil, a contrary current.*

" This is the cause of the winds that we observe. The little experiment which follows, and which is due to Franklin, very well represents what takes place in the atmosphere. Open in winter a door communicating between a hot and a cold room, there will be two currents: the one above, and directed from the hot to the cold apartment: the other below, and in a contrary direction.

" To be convinced of this, it is only necessary to place two tapers by the door, the one high up and the other low down; the flame of the former will be directed from within outwards, that of the latter in the contrary direction. Sometimes these two currents exist above and below a pane of glass when imperfectly puttied in; and we observe, in winter, that a thicker layer of ice is accumulated at its lower part. In a chimney and in a lamp-glass an ascending current is kept up, which feeds the flame; and this current is stronger as the sides of the chimney-funnel or the lamp-glass are more heated." 34.

The interesting phenomena of the morning sea-breeze and evening land-wind in tropical countries are readily explicable in a similar manner.

It is a curious but well-ascertained fact that, in the upper regions of the atmosphere within the tropics, there is almost always a westerly current of air: in the Northern hemisphere it is S.W., and in the Southern it is a N.W. wind. The heated air, that is continually rushing from the equatorial regions towards the poles, is directed westward by the rotatory motion of the earth. As the current arrives at the higher latitudes, it loses its velocity and heat, and descends about the 30th parallel of latitude. This is the origin of the S.W. winds, which prevail, even as far as the Pole, in the northern hemisphere—more especially over the Atlantic ocean. They blow with so much regularity, that the voyage from America to Europe is almost always much more rapid and easy than the return. The prevailing winds in Europe are S.W. and N.E.—the former arising in the manner which we have just mentioned; and the latter being produced by the current from the colder regions of the north-eastern regions of Russia towards the warmer regions of the south-western shores of our Continent.

In connexion with the subject of the *temperature* of the atmosphere, we may here introduce the following remarks on its *humidity*.

" In January, the coldest month of the year, the quantity of vapour attains its *minimum;* at the same time, the relative moisture is at its *maximum*. In proportion as the temperature rises, evaporation becomes more active, and the quantity of vapour increases, at first slowly, because the east winds, which commonly blow during this season, bring dry air from the interior of the continent. However, we must not deny, that the numbers for winter and spring differ probably much from means furnished by series embracing a greater number of years; for the latter winters have been warmer and the springs colder than they generally are. So that the numbers corresponding to winter are too high, and those to spring too low. The quantity of vapour attains its *maximum* in July, the month in which the air is driest. At the approach of winter, when the heat diminishes, the quantity of water precipitated in the form of rain, dew, and hoar-frost, greatly exceeds that which passes into the state of vapour. Its quantity, therefore, goes on diminishing, although the humidity is continually increasing, and is greater in November and December than in the month of January. This is the origin of the damp cold which characterises these two last months." 92.

Westerly winds are always, it is well known, much moister than easterly ones; the former come to us charged with vapour from the Atlantic, while the latter proceed from the interior of the continents of Europe and Asia.

*Weight of the Atmosphere; Cause of Storms, &c.*—Every one associates in his mind the Barometer with the idea of a weather-glass; and properly so, to a certain extent: because oscillations of this instrument, indicating as they do changes in the pressure of the atmosphere, usually precede or accompany changes in the weather. But let it be remembered that changes in the one are not necessarily or inevitably associated with corresponding changes in the other. The primary use of the Barometer is to point out the degree of the atmospheric pressure in the place where the observation is made, and its oscillations indicate certain changes in the amount of that pressure. Now, as these changes are almost always occasioned by the varying conditions of the atmospheric temperature in different columns of air, a secondary use of the instrument may be said to be to point out the differences of temperature between two places situated at considerable distances—just as a Differential Thermometer indicates the difference of temperature between two points very near together.

The following position therefore is one that suggests many curious considerations.

"*When the barometer falls in a country, it is because the temperature of this country is higher than that of the neighbouring countries, whether because it is heated directly or because these countries are cooled; on the contrary, the rise of the barometer proves that this country becomes colder than those which surround it.*" 263.

Keeping this principle in mind, we can understand how it is that, whenever the Barometer oscillates a great deal, the temperature and weather always experience extraordinary variations at some part or another of the globe—at a greater or less distance from the spot where the observations are made. A few examples, in illustration of this, may be interesting.

"About Christmas, in the year 1821, the barometer in Europe experienced an extraordinary fall. It was followed by a very mild winter in Paris, and in the other cities of west Europe; the mean temperature of January and February were higher by several degrees, than the general mean. In the United States, on the contrary, the winter was very severe; the current of the *Gulfstream* was directed towards places which it does not usually visit. In Persia, according to Fraser's account, the winter was very cold; as it was also in Africa, where the plains of Kordofan were covered with a bed of snow, which disappeared, it is true, very rapidly. At Paris, the following summer was drier and hotter by several degrees than usual. But, whilst the dry winds regained their sway in Europe, moist and violent winds constantly blew in India; at Bombay, 83,7 centimetres of rain above the mean fell; and in Kordofan, also, the Turkish army suffered much from continual rains.

"Something analogous occurred in 1824 and 1825. The terrible inundation of the Rhine in the autumn of 1838, the overflow of the Neva at St. Petersburg, the gales from the S.W., which, according to the observations of Munke and Schubler, took place in Schlesvig, and in all Holstein, are a proof of this. The barometer oscillated incessantly, and the rains were so abundant that every where, but principally in south Germany, springs burst forth in the streets and squares of the towns. In the same year the mean for the winter months was very high. In Iceland, on the contrary, according to Thorstensen's observations at Reikiavig, the mean of December was several degrees below the ordinary mean, and the barometer was very high, whilst it was very low at Copenhagen. This year, which was so rainy in Europe, was very dry in India; for at Bombay the quantity of rain was 118 centimetres below the mean. In Africa, it appears that there were violent gales; for, in the night of January 19th, 1825, the English ship *Clyde*, being 100 myriametres from the coast of Africa, was covered with fine sand, which the east winds had brought from the desert. In east Africa, Ruppel experienced violent storms: phenomena which are very rare in these countries, as may be discovered from the fear of the inhabitants. Ley experienced the same thing in Upper Egypt. In the following summer, all the north part of tropical Africa was a prey to excessive drought, and the inundation of the Nile having failed, there was a complete dearth.

"The same disturbances were manifested on the two shores of the Great Ocean. In California, there were violent tempests during the autumn, as there were also at the Sandwich and Phillippine Islands. But the trade-winds were no longer blowing regularly over the sea. These facts prove that the anormal phenomena of Europe are not isolated, but are propagated over the whole circumference of the globe." 323.

Besides these instances, we may mention that the winter of 1829-30 was one of the coldest that had occurred in Europe for a long time: yet, it was so mild in America that there was no ice on the west coast—a circumstance which permitted Captain Ross to advance so far to the north. The reverse of this was the case in the winter of 1833-4. It was unusually mild with us, and had been preceded by a stormy tempestuous autumn. In India, Brazil, and Guiana, the drought was so great that very many of the inhabitants died of famine. In China, there were terrible inundations; on the contrary, the rising of the Nile was quite insignificant. In the United States and in Persia the winter was extremely rigorous; and it was during this season that Captain Back encountered such terrible cold in the Polar regions.

The general conclusions, which Dr. Kraemtz has drawn from his researches on the subject, are these:—

"Thus, then, a great fall of the barometer, or frequent oscillations of the column, prove that there are meteorological disturbances on the surface of the globe, and conflicts of opposite winds, which change the weather. Further, when the barometer rises and falls rapidly, we may affirm that the weather will be variable for a long time. If we knew the weather that prevailed on the rest of the globe, we might conclude that which could be expected. We ought to know, when the barometer is low, whether the cold is very intense in America or in Asia. In the first case, the west winds will bring rain; in the second, the east winds will bring cold. However, on studying in the spring the barometer and the direction of the gales of wind, we may establish certain probabilities. If the barometer has fallen considerably during S.W. winds, and then rises slowly; if the wind passes from the West to the N.W., and remains in that direction; it is a proof of the predominance of west winds, and the weather will be influenced by them: we saw this in 1833. If the barometer, on the contrary, rises very quickly, and if the wind passes in a short space of time from the S.W. to the N.E., where it stops, we may expect a prolonged cold, like that which prevailed in 1829." 326.

In the Chapter on Atmospheric Electricity, there is the following description of what have been called *fulgurites*, or *fulminary tubes*.

"When lightning falls upon sand, its course is often marked by tubes, called fulminary tubes, or *fulgurites*. Although they have long been noticed, it is only since Henzen observed them in the sandy hillocks of Holstein that they have been attentively studied. Blumenbach was the first to attribute them to lightning; Fiedler carefully studied their nature and their mode of formation. They are generally composed of tubes of very different lengths and diameters, which contract toward their lower extremity, and terminate in a point; they are generally sinuous, and more or less ramified. Vitrified within, their exterior is covered with agglutinated grains of sand; the vitrified parts of which are of a reddish, or even greenish pearl grey. Their diameter is from 1 to $90^{mm}$; the thickness of the sides, $0^{mm},5$ to $24^{mm}$. Their length sometimes exceeds $6^{m}$, and the ramifications are from 2 to 30 centimetres long. All fulminary tubes with thick sides have, according to Fiedler, rough surfaces, and are divided into fragments of from 5 to $100^{mm}$ long. The tubes, whose sides are thin throughout their length, have a smooth surface, and are regularly cylindrical; they present no transverse fractures. All the fulgurites hitherto examined are directed towards reservoirs of water, or bodies which are good conductors of electricity.

"Direct observations have shewn that these fulgurites were due to the action of lightning. Thus, Pfaff received a tube from the island of Amrum. Some sailors saw the lightning fall upon the sand; they dug and found a tube $6^{mm}$ in diameter, blackened within by the carbon of the burned vegetables." 355.

The *Heat-lightnings*, that are so frequently observed on serene evenings in summer, are regarded by most meteorologists, and by our author among the number, as the reflections of the lightning of distant storms. "Every one," says he, "may convince himself that lightnings are reflected through the air with great intensity on a dark night. When a storm is in the west, and the rest of the sky remains serene, we have only to turn our back to the storm to see the lightning reflected in the east part of the heavens; and yet, in this case, the conditions for reflection are far less favourable than in the preceding example."

It is to be observed, that, whenever these *heat*-lightnings are more than usually frequent and brilliant, the days are generally heavy and dull, betokening the approach of a storm, and the Barometer indicates a tendency either to rise or fall. In the majority of instances, we shall find that a severe storm has been experienced at a greater or less distance from the spot where the phenomena were observed. For example:—

"In the middle of the month of August, 1832, heat-lightning was very frequently observed at Geneva; during the day the sky had a dull and dim appearance. On August 16, the subject was actively discussed in the *Societé de Physique et d'Histoire Naturelle*. After the sitting, heat-lightning illuminated the whole of the north horizon, as though to put to the test the opinions that had been advanced. During the day the sky had been dull; toward the north I had perceived *cumulo-stratus* about the horizon; and after sunset there still remained clouds in the sky. Some days after, the newspapers were filled with the account of ravages caused by storms in Baden, Wurtemberg, and Bavaria: even in the country of Vaud, the lightning struck several houses. If the newspapers had also mentioned the storms that had occurred in this neighbourhood, without causing mischief, we might have seen that there were some even closer still." 375.

Among the electrical phenomena of the atmosphere our author enumerates *hail*—the formation of which meteor has long been, and still remains, a subject of difference of opinion. How, for instance, are we to explain the circumstance that hail-storms are infinitely more common during the day than during the night? and that, in the majority of instances, they are observed in warm weather, at, or soon after, noon, about the period of the greatest diurnal heat? Then again, how comes it that certain places are ravaged by hail-storms almost every year, whilst adjacent localities are almost entirely spared? These, and many other points connected with the history of this atmospheric phenomenon, are far from being well understood.

Aristotle and Lucretius have said that a loud noise may often be heard when a cloud, charged with hail, approaches the zenith. This observation has been confirmed by several modern writers; while others have either questioned, or wholly denied, its accuracy. As we have already mentioned, hail-showers are frequently very limited in their extent: at a short distance from where the hail fell, perhaps not even a puff of wind has been felt. The following is a curious example of this circumstance.

"The storm commenced in the morning in the south of France, and in a few hours reached Holland. The places destroyed by the hail formed two parallel lines from S.W. to N.E.; one was 70 and the other 80 myriametres long. The mean width of the west line was 16, and that of the east line 8 kilometres. The space comprised between the two lines, the width of which was 2 myriametres, was spared; there merely fell a heavy rain. It also rained much on the east and the west of the two lines. The storm was preceded by an obscuration of the light of day; it travelled about 66 kilometres per hour, and its velocity was the same in both the zones. In the west zone the hail fell at La Rochelle, after a storm that had lasted all the night; at $17^h$ $30^m$, in Touraine, near Loches; at $18^h$ $30^m$, near Chartres; at $19^h$ $30^m$, at Rambouillet; at $20^h$, at Pontoise; at $20^h$ $30^m$, at Clermont, in Beauvoisis; at $21^h$, at Douai; at $23^h$, at Courtrai; at $0^h$ $30^m$, and at Flessingue, about $1^h$ $30^m$. In the west zone, the storm reached Artenay, near Orleans, at $19^h$ $30^m$; Andouville, in La Beauce, about $20^h$; the Faubourg St. Antoine, Paris, $20^h$ $30^m$; Crespy, in Valois, about $21^h$ $30^m$; Cateau-Cambresis, $23^h$; Utrecht, $2^h$ $30^m$. At each place the hail only fell for seven or eight minutes, but with so much violence, that all the harvests were cut to pieces. Of all the great hail-storms, there is not one of which we have such exact information, and yet it is still insufficient; thus the direction of the wind, and that of the clouds before and after the storms, and on the two sides of the space where the hail fell, have not been pointed out." 384.

According to Dr. Kraemtz, the production of hail—which, it is to be observed, is almost invariably preceded by oscillations in the barometric state of the atmosphere—is attributable to the conflict of two opposing currents of wind from the north and south, (the latter charged with moisture)—the greatest fall taking place where the shock is the most violent. After a hail-storm, the weather—which usually has been hot for some time before—often remains unsettled for weeks; very frequently it is followed by cold. The rise of the barometer proves that the north wind generally obtains the predominance; and the more so, as, in melting, the hail absorbs a very notable quantity of heat.

" The conflict of opposite winds," our author remarks, " also explains certain peculiarities of storms, accompanied by hail. Every thing that tends to put the air in motion favours the formation of hail. This is why it is more common in the mountains, where the rapidity of the atmospheric current increases in the valleys. If the march of storms were known by observations embracing a series of several years, by comparing local peculiarities, we might discover why certain countries are frequently ravaged by hail, whilst others are almost entirely spared. Narrow valleys, surrounded by high mountains, as the Vallais and the vale of Aoste, are rarely visited by hail: these valleys are so warm, that the hailstones melt before reaching the ground. Moreover, the high mountains that hang over them prevent the conflict of opposite winds, or limit it to the high regions of the atmosphere, which prevents the hailstones acquiring any considerable volume. But, at the mouth of valleys, in the plain, storms, accompanied with hail, are the more violent (principally on the south side of the Alps), as the south winds are arrested by the mountains, while the north winds, when they have traversed them, rush impetuously to the plain." 391.

With the following quotation on the curious subject of *Terrestrial Magnetism*, we close our notice of this interesting volume.

" Every where, on the surface and in the interior of the globe, the magnetic needle takes a determinate position; this position varies in each country of the earth: as we advance, proceeding directly to the west, we see that it increases, and attains its *maximum* in the Atlantic Ocean. From this point, the western declination diminishes; and at the east of the United States, the needle points exactly to the north pole, and consequently the declination is nothing; more westward, the declination becomes east. If we had gone toward the east, the west declination would have diminished; it would be nothing in the east of the Russian empire; and then east, if we had continued our course toward the east.

" In general, if, under any parallel, we take the circuit of the globe, we shall find a point where the needle is directed toward the north; afterwards the deviation becomes westerly, attains its *maximum*, and then diminishes, until it is nothing: thus the declination varies greatly. If we make our experiments under the equator, and repeat them every five degrees, we shall find that the difference between the *maximum* eastern and the *maximum* western declination increases as we approach the poles of the earth. Thus, in Greenland, the west declination is so great that the needle points to the west; and Parry found a point, in the west of Greenland, where the north pole of the needle was turned to the south.

" The dip presents similar differences; in our countries it is northern, that is to say, the north pole is directed downward, and forms an angle of 70° with the plane of the horizon. In proportion as we advance toward the south, the needle approaches the horizontal direction, and, in the neighbourhood of the equator, it is altogether parallel to the horizon: the dip is, therefore, nothing. On passing into the southern hemisphere, we see the south pole of the needle dip, and the more so as we approach the south pole; going toward the north, the contrary would have been observed: thus, in one of the hemispheres, the dip is northern, and, in the other southern. These two hemispheres are separated by a line of dip, upon all points of which the needle is horizontal: this line, which cuts the equator in different points, and rises alternately into each hemisphere, is called the *magnetic equator*." 548.

